# A novel homozygous variant in *C1QBP* causes severe IUGR, edema, and cardiomyopathy in two fetuses

**DOI:** 10.1002/jmd2.12209

**Published:** 2021-03-05

**Authors:** Morten Alstrup, Ida Vogel, Puk Sandager, Jenny Blechingberg, Naja Becher, Elsebet Østergaard

**Affiliations:** ^1^ Department of Clinical Genetics Copenhagen University Hospital Rigshospitalet Copenhagen Denmark; ^2^ Center for Fetal Diagnostics, Department of Clinical Medicine Aarhus University Hospital Aarhus Denmark; ^3^ Department of Clinical Genetics Aarhus University Hospital Aarhus Denmark; ^4^ Department of Obstetrics and Gynecology Aarhus University Hospital Aarhus Denmark

**Keywords:** C1QBP, cardiomyopathy, IUGR, mitochondrial, prenatal phenotype

## Abstract

The C1QBP protein (complement component 1 Q subcomponent‐binding protein), encoded by the C1QBP gene, is a multifunctional protein predominantly localized in the mitochondrial matrix. Biallelic variants have previously been shown to give rise to combined respiratory‐chain deficiencies with variable phenotypic presentation, severity, and age at onset, from intrauterine with a mostly lethal course, to a late‐onset mild myopathy. We present two fetuses, one male and one female, of first‐cousin parents, with severe intrauterine growth retardation, oligo/anhydramnios, edema, and cardiomyopathy as the most prominent prenatal symptoms. Both fetuses showed no copy number variants by chromosome microarray analysis. Analysis of a fibroblast culture from one of the fetuses showed deficiency of respiratory chain complex IV, and using exome sequencing, we identified homozygosity for a novel variant in *C1QBP* in both fetuses. To our knowledge, only six patients with pathogenic variants in *C1QBP* have been reported previously and with this report, we add a novel pathogenic variant in *C1QBP* found in two related fetuses.

**SYNOPSIS**

Homozygosity for a novel variant in *C1QBP* in two fetuses cause intrauterine disease onset with intrauterine growth retardation (IUGR), oligo/anhydramnios, and cardiomyopathy, with lethal preterm outcome.

## 1 INTRODUCTION

Mitochondria are an essential part of eukaryotic cells, and their primary function is ATP production via the oxidative phosphorylation pathway.^1^ Mitochondrial diseases are a class of heterogeneous diseases, mainly affecting organs with high energy demands, that cause a wide range of phenotypical and mild to severe symptomatic presentations in patients with single or multisystemic affected organs.^1^, ^2^ Next‐generation sequencing has given the opportunity to identify nuclear and mitochondrial DNA gene defects causing mitochondrial diseases, and approximately 250 disease‐associated genes have been described.^2^, ^3^, ^4^, ^5^

*C1QBP* (MIM *601269) encodes the complement component 1 Q subcomponent‐binding protein (C1QBP), which is a multifunctional protein predominantly localized in the mitochondrial matrix.^5^ *C1QBP* has been shown to be involved in mitochondrial ribosome biogenesis, regulation of apoptosis, and infectious and inflammatory processes.^3^, ^6^, ^7^ Dysfunctional C1QBP has been shown to cause combined respiratory chain deficiency due to severely impaired mitochondrial protein synthesis, leading to midgestational lethality, impaired intrauterine development, growth retardation, postnatal cardiac dilatation, contractile dysfunction, and cardiac fibrosis in mice.^5^, ^8^

In a recent study, pathogenic biallelic variants in *C1QBP* were identified in four unrelated patients, causing a combined respiratory‐chain enzyme deficiency, termed combined oxidative phosphorylation deficiency 33 (COXPD33, MIM #617713), an autosomal recessive mitochondrial disease. Disease onset was variable, from intrauterine to adulthood. The phenotypic presentation varied from infantile lactic acidosis to childhood myopathy and late‐onset myopathy with progressive external ophthalmoplegia (PEO). However, all subjects showed cardiomyopathy.^3^

In this paper, we report a family of consanguineous parents from Syria with two fetuses with a previously unreported variant in *C1QBP* in a homozygous state. Both fetuses' phenotypic presentation included severe IUGR, oligo/anhydramnios, cardiomyopathy with cardiac effusion, and later hydrops foetalis.

### 1.1 Case presentation

The couple are first cousins from Syria. Subject 1 (S1) and subject 2 (S2) were their first and third pregnancy, respectively. Their second child from the second pregnancy is a healthy boy.

**FIGURE 1:**
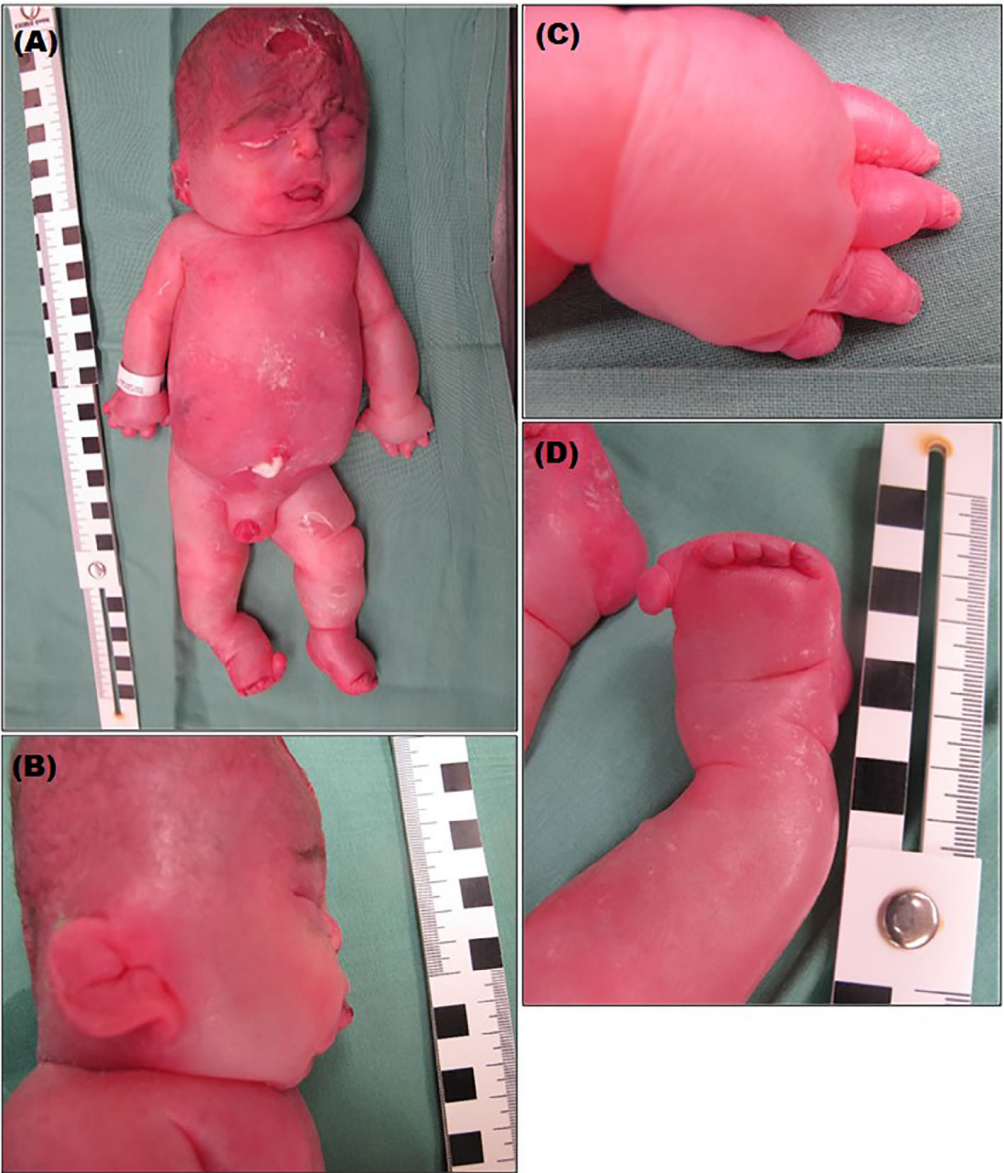
Postmortem clinical and morphologic features of S1 with (A) and (B) showing severe growth restriction, dysmorphic facial findings, ascites, generalized skin edema, and hydrops foetalis. (C) and (D) showing extremity growth restriction, edema, sandal gap toes and talipes equinovarus congenita

S1 was a male, where the combined first trimester screening (cFTS) was normal. An ultrasound scan at 20 weeks of gestation showed a small fetus with head circumference (HC) and abdominal circumference (AC) around −4 SD, oligohydramnios, and reduced fetal movements were noted. An amniocentesis was performed at 20 + 2 weeks of gestation. The fetal MRI of the cerebrum performed at 21 weeks of gestation, showed no abnormalities and a TORCH test was negative. Subsequent ultrasound scans showed additional pathological findings, including severe IUGR, extremity growth restriction, and echogenic bowel, as well as an enlarged fetal heart with pericardial effusion, biventricular dysfunction and frequently occurring episodes of bradycardia. From 28 to 33 weeks of gestation, the disease progressed with progression of dilated cardiomyopathy and reduced biventricular function, pulmonary hypoplasia, anhydramnios, and hydrops foetalis with ascites, and generalized skin edema (Figure 1). At 33 weeks of gestation, S1 was stillborn with a birthweight of 1716 g. A postnatal autopsy revealed, in addition to antenatal ultrasound findings, a large placenta with villous edema, large quantities of immature erythrocytes in the fetal circulation, focal dermal bullae, dysmorphic facial findings, talipes equinovarus congenita, sandal gap toes, hydrothorax, and hepatomegaly (Figure 1).

S2 was a female, where the cFTS at 13 + 1 weeks of gestation showed an increased nuchal translucency of 3.7 mm, abnormal flow in the ductus venosus with negative a‐wave and choroid plexus cysts were suspected. A chorion villus sample was taken at 13 + 2 weeks of gestation. At 15 weeks of gestation, the HC and AC were around −2 SD, and a cardiac defect, ventricular septum defect, was suspected. This was confirmed in week 17, where furthermore, abnormal contractility of the heart, pericardial effusion, and frequent episodes of bradycardia were noted. At 19 + 5 weeks of gestation, HC and AC were −3.3 SD, and the myocardium was hyperechogenic. The pregnancy was terminated at 20 weeks of gestation. The fetal birth weight was 170 g. A postnatal autopsy revealed, in addition to the antenatal ultrasound findings, a large cyst bordering a dilated fourth ventricle, cerebellar hypoplasia, and multiple small periventricular cerebral hemorrhages in an otherwise normally developed cerebrum. Facial dysmorphisms, extremity contractures, sandal gap toes, underdeveloped ovaries, and a mediastinal cystic hygroma were also found. The placenta was small and hypoperfused.

### 1.2 Genetic and biochemical analyses

Analysis of DNA extracted from amniotic cells from S1 and chorionic villus sampling from S2 showed no copy number variants using chromosomal microarray (Agilent 180K oligo array). Exome sequencing was performed using DNA from both fetuses and the parents. The Nimblegen MedExome kit (Roche, Basel, Switzerland) was used for capture, and sequencing was done on a NextSeq 500 (Illumina, San Diego, California). The mean sequencing depth was 124×, with a sequencing depth of >20× for 96% of the targeted region. Data analysis was performed using an in‐house GATK‐pipeline, and data were filtered using Ingenuity (QIAGEN, Hilden, Germany). Delly and ExomeDepth was used for copy‐number analysis. Autosomal recessive inheritance was assumed, whereas no phenotype data was used in the filtering of data. A previously unreported variant in *C1QBP*, c.743T>C (reference transcript NM_001212.3), was found in a homozygous state in DNA from both fetuses, and in a heterozygous state in both parents. The variant was absent from gnomAD (https://gnomad.broadinstitute.org/) and predicted to lead to an exchange of a highly conserved valine with an alanine, p.(Val248Ala) (Table 1; UCSC Genome browser). The CADD score was 27.3.^9^

**TABLE 1:** Conservation of the affected valine amino acid across different species. Alignment of homologs are shown in bold

Human	248	D	H	L		M	D	F	L	A	D	R	G	V	D	N	T	F	A	D	E	L	V	E	L	S
Mutated	248						D	F	L	A	D	R	G	**A**	D	N	T	F	A	D	E	L	V	E	L	
*Pan troglodytes*	248						D	F	L	A	D	R	G	**V**	D	N	T	F	A	D	E	L	V	E	L	
*Macaca mulatta*	247						D	F	L	A	D	R	G	**V**	D	N	T	F	A	D	E	L	V	E	L	
*Mus musculus*	245						D	F	L	A	D	R	G	**V**	D	N	T	F	A	D	E	L	V	E	L	
*Gallus gallus*	143	D	H	L		M	D	F	L	A	D	R	G	**V**	D	N	T	F	A	D	E	L	I	E	L	
*Danio rerio*	235	D	H	L		M	D	F	L	A	D	R	G	**V**	D	N										
*Drosophila melanogaster*	228	D	L	L		I	N	L	L	E	E	K	G	**I**	S	Q	E	F	A	E	K	L	S	D	L	
*Caenorhabditis elegans*	202	D	L	L	F	V	R	Y	L	E	E	R	G	**L**	D	A	R	F	C	K	T	L	V	A	Y	
*Xenopus tropicalis*	250	D	H	L		M	D	F	L	A	D	R	G	**V**	D	N	T	F	A	D	E	L	V	E	L	

**FIGURE 2:**
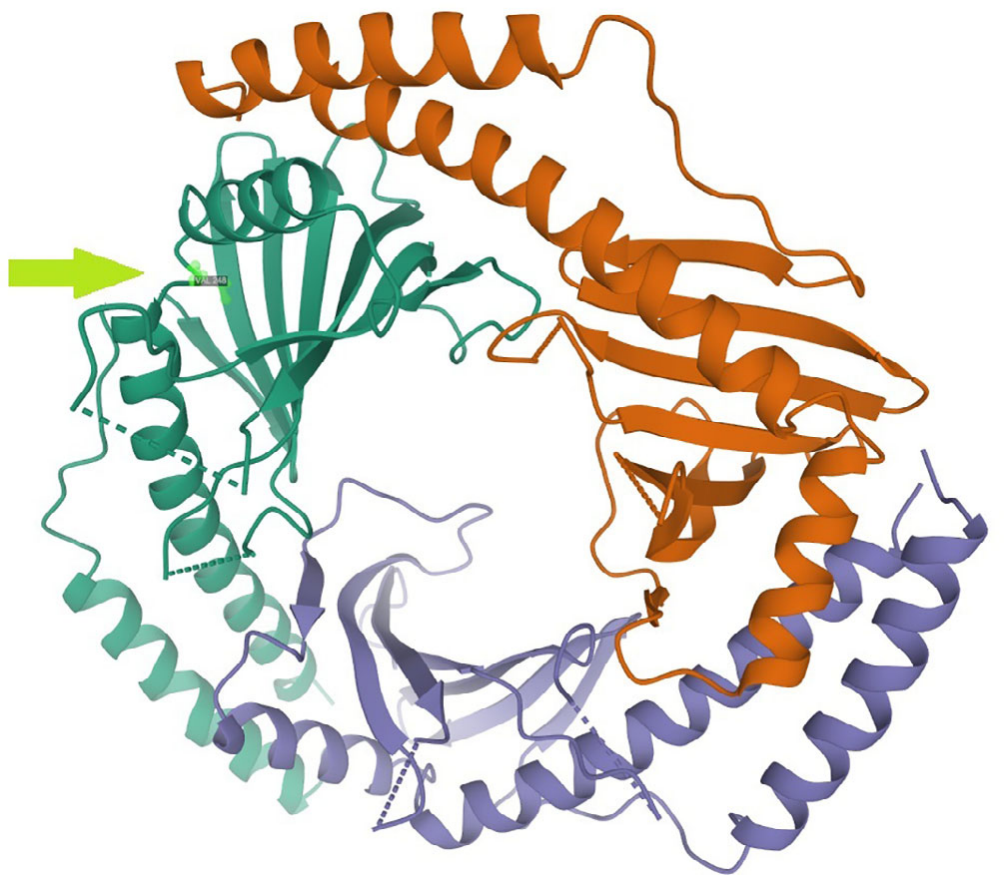
The protein structure was performed with Mol* (RSCB PDB: 1P32). Highlighting the affected amino acid valine at position 248, localized on a hydrogen‐bonded turn of the C1QBP protein

C1QBP forms a doughnut‐shaped homotrimer with an unusually asymmetric charge distribution on the surface (Figure 2). The affected amino acid, valine at position 248, is localized in an important structural domain of the protein, on the hydrogen‐bonded turn of the protein, adjacent to another disease‐causing variant, p.Gly247Trp^3^ (RCSB Protein Data Bank).

A fibroblast culture from S2 was used for analysis of respiratory chain enzyme activity, performed essentially as described previously.^10^ Analysis of complexes II, III, and IV showed a decreased complex IV activity of 0.7 (reference range, 1.2‐3.2), that is, 58% of lowest control, whereas the activities of complex II (0.57 [reference range, 0.38‐0.76]) and III (1.6 [reference range, 1.0‐1.8]) were within the reference range. The values were normalized to citrate synthase.

## 2 DISCUSSION

Here, we report two fetuses from a first‐cousin couple, homozygous for a novel variant in *C1QBP*. We believe that the variant is disease‐causing based on the following: (a) the variant segregated with the disease in the family, (b) it was scored damaging or pathogenic by 16/20 in silico analyses,^11^ (c) it was associated with decreased activity of complex IV in fibroblasts, (d) the affected amino acid is highly conserved across different species (Table 1; Figure 2), and finally (e) the phenotype is in concordance with that of other patients with pathogenic variants in *C1QBP*. The main clinical findings in the two fetuses reported here were severe IUGR, oligo/anhydramnios, and cardiomyopathy.

To our knowledge, only six patients with pathogenic variants in *C1QBP* have been reported. An earlier study of four patients with biallelic *C1QBP* variants demonstrated varying clinical manifestations with onset of disease from prenatal to 57 years of age. All four patients presented with cardiac symptoms, presumably causing two neonatal deaths, but associated with a relatively stable course in patients with later presentation.^3^ A mouse study showed that systemic knockout and genetic ablation of *C1QBP* caused cardiac dilation, cardiac fibrosis, and contractile dysfunction, which supports the importance of *C1QBP* for cardiac function.^8^ However, in a recent study, two unrelated adult patients homozygous for a variant in *C1QBP* had no signs of cardiomyopathy.^12^ Both patients developed ptosis in adulthood (28 and 45 years, respectively) and were diagnosed with late onset progressive external ophthalmoplegia (PEO), and slowly developed reduction of ocular motility, myopathy, and hyposthenia. There was no evidence of central nervous system involvement. Individual 3 and 4 in the study by Feichtinger et al. were also diagnosed with late onset PEO as the initial symptom, and cardiomyopathy found later on follow‐up.^3^

The two fetuses reported here share phenotypic presentations with individual 1 and 2 from the study by Feichtinger et al,^3^ namely IUGR, oligo/anhydramnios, generalized edema, cardio/hepatomegaly, cortical hemorrhages, and preterm birth within 20 to 34 weeks of gestation. Studies of other mitochondrial diseases caused by pathogenic variants in, for example, *ACAD9*, *POLG*, *COQ9*, and *FBXL4*, have shown prenatal onset of mitochondrial disease with intrauterine growth retardation, lactic acidosis, cardiomegaly, anemia, renal abnormalities, dysmorphic facial features, cerebellar hypoplasia and often preterm, or shortly thereafter, fatality.^13^, ^14^, ^15^, ^16^ However, the large variability in phenotypic presentation, the large number of genes involved in mitochondrial function, and the unknown prenatal phenotype for most mitochondrial diseases highlights the utility of exome/genome sequencing in the diagnostic workup.

The variability in phenotypic presentations of the eight different patients diagnosed with pathogenic variants in *C1QBP* could possibly be explained by the different localizations of affected amino acids, as previously suggested.^3^, ^12^ The variants associated with cardiomyopathy (p.Cys186Ser, p.Leu275Pro, p.Leu257Phe, and p.Gly247Trp [Feichtinger et al]) are localized in important structural areas of the protein (fifth beta strand, alpha C helix, and on hydrogen‐bonded turn, respectively); this also applies to the variant reported here (p.Val248Ala) which is localized on the hydrogen‐bonded turn^6^ (Figure 2). In contrast, two patients with the p.Tyr188del variant and one with the p.Phe204Leu variant,^3^, ^12^ localized in the less important coiled‐coil region^6^ showed a milder phenotype with later onset PEO. Thus, although C1QBP is important for cardiac function, variants in *C1QBP* seem to be also associated with a milder PEO phenotype without (early) cardiomyopathy.

Analysis of a fibroblast culture from one of the fetuses reported here showed deficiency of respiratory‐chain (RC) complex IV, which is in accordance with previous report,^3^ whereas analyses of muscle and liver homogenates in postnatal cases have shown a combined deficiency of RC complexes I, III, and IV. A combined respiratory chain enzyme deficiency was also found in a muscle and liver from knockout mice.^5^ The combined RC enzyme deficiency is in line with the presumed role of *C1QBP* in mitochondrial protein synthesis, however *C1QBP* is also assumed to play a role in mitochondrial genome maintenance, illustrated by the finding of multiple mtDNA deletions in patients with a primary PEO phenotype.

In summary, we identified homozygosity for a novel variant in *C1QBP* in two fetuses of first‐cousin parents. Disease onset was intrauterine with IUGR, oligo/anhydramnios, and cardiomyopathy as the most prominent signs, both with lethal preterm outcome. Eight patients have been reported with pathogenic variants in *C1QBP*, and the localization of the variants potentially correlates with the development of either an early, potentially lethal, phenotype with major cardiomyopathy, or later onset PEO.

## CONFLICT OF INTEREST

The authors declared no potential conflicts of interest.

## AUTHOR CONTRIBUTIONS

Morten Alstrup: drafting of the manuscript. Ida Vogel, Puk Sandager, Jenny Blechingberg, Naja Becher: contributed with data collection, clinical information and thorough analyses of collected data. Elsebet Østergaard: critical revising of the manuscript, approved the final version. All authors critically reviewed and approved the final version of the manuscript.

## INFORMED CONSENT

All procedures followed were in accordance with the ethical standards of the responsible committee on human experimentation (institutional and national) and with the Helsinki Declaration of 1975, as revised in 2000. Informed consent was obtained from all patients for being included in the study. This article does not contain any studies with animal subjects performed by the any of the authors.
